# Use of ultrasound elasticity imaging to monitor the response of a primary breast cancer to neoadjuvant chemotherapy in one patient in a pilot study

**DOI:** 10.1186/bcr2995

**Published:** 2011-11-04

**Authors:** RE English, A Di Battista, RF Adams, L Winter, JA Noble

**Affiliations:** 1 Oxford Radcliffe Hospitals NHS Trust, Oxford, UK; 2 Oxford University, Oxford, UK

## Introduction

Neoadjuvant chemotherapy is used as the primary treatment for locally advanced breast cancer to reduce tumour size and protect against metastatic spread. Monitoring the response to chemotherapy is achieved mainly by clinical assessment, which may prove unsatisfactory. This proof-of-principle study is to determine if changes of size and texture in the elastogram can track tumour response.

## Methods

A young woman with an invasive ductal carcinoma, visible on mammography and ultrasound (US), was treated with seven cycles of chemotherapy. Diagnostic mammography, MRI and core biopsy studies were performed. B-mode US and elastography datasets were collected in two planes prior to each cycle using a coarse calcification within the tumour as a constant reference point. Seven sequential strain images, each 3 weeks apart, were collected in total. The ratio of tissue strain within and outside the mass was calculated as an indicator of tumour response. The findings were compared with the post-chemotherapy MRI and mastectomy histology.

## Results

The tumour showed almost complete histological response to chemotherapy. Sequential elastography studies demonstrated significant ratio changes in stiffness in tumour and peritumoral areas.

## Conclusion

Preliminary results suggest the changes in tumour and peritumour stiffness caused by chemotherapy are detectable by ultrasound elastography. Further investigation is required to evaluate the potential of elastography as a monitoring tool for chemotherapy treatment.

